# Supplementary material for: Multi‐dataset identification of innovative feature genes and molecular mechanisms in keratoconus
Source: J Cell Mol Med. 2024 Sep 19;28(18):e70079. doi: 10.1111/jcmm.70079 (PMC11412914; doi:10.1111/jcmm.70079)
Supplement: Supplementary file 2 — Material S2. [file JCMM-28-e70079-s002.zip › 7.GSEA analysis/6.SNORA74B/KEGG_RNA_DEGRADATION.html]

Details for gene set KEGG\_RNA\_DEGRADATION[GSEA]

|  || Dataset | SNORA74B.group.cls#h\_versus\_l |
| Phenotype | group.cls#h\_versus\_l |
| Upregulated in class | h |
| GeneSet | KEGG\_RNA\_DEGRADATION |
| Enrichment Score (ES) | 0.1825944 |
| Normalized Enrichment Score (NES) | 0.96562946 |
| Nominal p-value | 0.5734694 |
| FDR q-value | 1.0 |
| FWER p-Value | 1.0 |
Table: GSEA Results Summary

  

Fig 1: Enrichment plot: KEGG\_RNA\_DEGRADATION      
 Profile of the Running ES Score & Positions of GeneSet Members on the Rank Ordered List

  

| SYMBOL | TITLE | RANK IN GENE LIST | RANK METRIC SCORE | RUNNING ES | CORE ENRICHMENT || 1 | DIS3 | na | 3858 | 0.264 | -0.0218 | Yes |
| 2 | EXOSC6 | na | 5823 | 0.198 | -0.0115 | Yes |
| 3 | CNOT1 | na | 7012 | 0.162 | 0.0090 | Yes |
| 4 | PATL1 | na | 10488 | 0.083 | -0.0629 | Yes |
| 5 | DDX6 | na | 10689 | 0.080 | -0.0416 | Yes |
| 6 | ZCCHC7 | na | 10850 | 0.077 | -0.0200 | Yes |
| 7 | CNOT2 | na | 11185 | 0.072 | -0.0054 | Yes |
| 8 | CNOT6 | na | 11986 | 0.060 | -0.0081 | Yes |
| 9 | PAPOLB | na | 12154 | 0.058 | 0.0066 | Yes |
| 10 | CNOT6L | na | 12232 | 0.057 | 0.0236 | Yes |
| 11 | DCPS | na | 12382 | 0.055 | 0.0378 | Yes |
| 12 | PAPOLA | na | 12444 | 0.054 | 0.0543 | Yes |
| 13 | EXOSC3 | na | 13988 | 0.036 | 0.0220 | Yes |
| 14 | CNOT10 | na | 14124 | 0.034 | 0.0297 | Yes |
| 15 | ENO1 | na | 15810 | 0.017 | -0.0133 | Yes |
| 16 | CNOT3 | na | 16220 | 0.012 | -0.0209 | Yes |
| 17 | EXOSC4 | na | 16393 | 0.010 | -0.0223 | Yes |
| 18 | EXOSC1 | na | 16542 | 0.009 | -0.0236 | Yes |
| 19 | LSM7 | na | 16629 | 0.008 | -0.0233 | Yes |
| 20 | EXOSC9 | na | 16903 | 0.005 | -0.0293 | Yes |
| 21 | LSM3 | na | 17430 | 0.000 | -0.0444 | Yes |
| 22 | DCP2 | na | 18062 | -0.007 | -0.0603 | Yes |
| 23 | EXOSC2 | na | 18131 | -0.007 | -0.0598 | Yes |
| 24 | DCP1A | na | 18135 | -0.007 | -0.0573 | Yes |
| 25 | PARN | na | 18282 | -0.009 | -0.0584 | Yes |
| 26 | ENO3 | na | 18355 | -0.010 | -0.0570 | Yes |
| 27 | XRN1 | na | 18544 | -0.012 | -0.0583 | Yes |
| 28 | LSM8 | na | 19039 | -0.017 | -0.0666 | Yes |
| 29 | LSM1 | na | 19056 | -0.018 | -0.0611 | Yes |
| 30 | EDC3 | na | 19307 | -0.021 | -0.0613 | Yes |
| 31 | DCP1B | na | 19433 | -0.022 | -0.0574 | Yes |
| 32 | EXOSC8 | na | 19830 | -0.027 | -0.0598 | Yes |
| 33 | C1D | na | 19869 | -0.027 | -0.0518 | Yes |
| 34 | LSM5 | na | 19937 | -0.028 | -0.0443 | Yes |
| 35 | EDC4 | na | 20772 | -0.037 | -0.0557 | Yes |
| 36 | HSPD1 | na | 20854 | -0.038 | -0.0451 | Yes |
| 37 | CNOT7 | na | 21515 | -0.046 | -0.0486 | Yes |
| 38 | LSM4 | na | 21745 | -0.049 | -0.0387 | Yes |
| 39 | PNPT1 | na | 21812 | -0.049 | -0.0239 | Yes |
| 40 | LSM2 | na | 22026 | -0.052 | -0.0124 | Yes |
| 41 | EXOSC10 | na | 22392 | -0.057 | -0.0038 | Yes |
| 42 | LSM6 | na | 22541 | -0.059 | 0.0118 | Yes |
| 43 | XRN2 | na | 22639 | -0.060 | 0.0294 | Yes |
| 44 | PAPOLG | na | 23311 | -0.070 | 0.0337 | Yes |
| 45 | EXOSC7 | na | 24285 | -0.085 | 0.0345 | Yes |
| 46 | HSPA9 | na | 24653 | -0.092 | 0.0551 | Yes |
| 47 | CNOT4 | na | 25869 | -0.114 | 0.0587 | Yes |
| 48 | EXOSC5 | na | 26270 | -0.122 | 0.0885 | Yes |
| 49 | ENO2 | na | 26364 | -0.124 | 0.1279 | Yes |
| 50 | CNOT8 | na | 27750 | -0.158 | 0.1415 | Yes |
| 51 | MPHOSPH6 | na | 28396 | -0.176 | 0.1826 | Yes |
Table: GSEA details [plain text format]

  

Fig 2: KEGG\_RNA\_DEGRADATION      
 Blue-Pink O' Gram in the Space of the Analyzed GeneSet

  

Fig 3: KEGG\_RNA\_DEGRADATION: Random ES distribution      
 Gene set null distribution of ES for **KEGG\_RNA\_DEGRADATION**
